# Physical activity in adolescents with psychiatric disorders and in the general population

**DOI:** 10.1186/1753-2000-8-2

**Published:** 2014-01-22

**Authors:** Wenche Langfjord Mangerud, Ottar Bjerkeset, Stian Lydersen, Marit Sæbø Indredavik

**Affiliations:** 1 Regional Centre for Child and Youth Mental Health and Child Welfare, Faculty of Medicine, Norwegian University of Science and Technology (NTNU), Trondheim, Norway; 2 Faculty of Health Sciences, Nord-Trøndelag University College (HiNT), Levanger, Norway; 3 Department of Neuroscience, Faculty of Medicine, NTNU, Trondheim, Norway; 4 Department of Child and Adolescent Psychiatry, St. Olav’s University Hospital, Trondheim, Norway

**Keywords:** Physical activity, Prevalence, Sports, Psychiatric disorders, Adolescents

## Abstract

**Background:**

Adults who suffer from psychiatric disorders report low levels of physical activity and the activity levels differ between disorders. Less is known regarding physical activity across psychiatric disorders in adolescence. We investigate the frequency and type of physical activity in adolescent psychiatric patients, compared with adolescents in the general population.

**Methods:**

A total of 566 adolescent psychiatric patients aged 13–18 years who participated in the CAP survey, Norway, were compared to 8173 adolescents aged 13–19 years who participated in the Nord-Trøndelag Health Study, Young-HUNT 3, Norway. All adolescents completed a questionnaire, including questions about physical activity and participation in team and individual sports.

**Results:**

Approximately 50% of adolescents with psychiatric disorders and 25% of the population sample reported low levels of physical activity. Within the clinical sample, those with mood disorders (62%) and autism spectrum disorders (56%) were the most inactive and those with eating disorders (36%) the most active. This pattern was the same in individual and team sports. After multivariable adjustment, adolescents with a psychiatric disorder had a three-fold increased risk of lower levels of physical activity, and a corresponding risk of not participating in team and individual sports compared with adolescents in the general population.

**Conclusions:**

Levels of physical activity were low in adolescent psychiatric patients compared with the general population, yet activity levels differed considerably between various disorders. The findings underscore the importance of assessing physical activity in adolescents with psychiatric disorders and providing early intervention to promote mental as well as physical health in this early stage of life.

## Background

About one third of adolescents worldwide meet the criteria for a lifetime psychiatric disorder. Girls have higher rates of mood and anxiety disorders, while boys have higher rates of behavioral disorders [1]. Several cross-sectional studies report an association between certain psychiatric disorders and reduced levels of physical activity in adults [2,3]. Physical inactivity has a major negative impact on public health [4], and has been identified as the fourth leading risk factor for non-communicable diseases, accounting for many premature and preventable deaths [5]. Furthermore, physical activity in childhood and adolescence might serve as a predictor for the level of physical activity later in life [6]. Generally, boys participate in more physical activity than girls and the level of physical activity declines during the teenage years [7,8]. Adolescents from families with higher socioeconomic status (SES) are more physically active than those with lower SES, yet these findings remain somewhat unclear [9].

In a large cross-sectional study of about 2500 British adolescents, lower levels of physical activity were associated with more mental health problems than higher levels of physical activity [10]. In contrast, adolescents with eating disorders reported high levels of physical activity, called “driven exercise”, in an American cross-sectional study [11]. In a review, those adolescents with binge eating disorder tended not to exercise at all [12]. Children with attention-deficit hyperactivity disorder (ADHD) showed increased levels of physical activity in general in a large German cross-sectional study, but were less likely to engage in organized sports [13].

In several cross-sectional studies adolescents who engaged regularly in physical activity reported lower anxiety-depression scores than those who were less active [14,15]. In a review, researchers concluded that physical activity in psychiatric patients may reduce psychological symptoms [16].

Participation in individual sports and team sports is associated with several factors, including sex, age and SES [17]. Also, adolescents with depressive symptoms, are suggested to be less likely to participate in team sports [18], while boys with conduct disorder frequently participate in team sports [19]. Some studies have found more athletes with eating problems in individual sports like ballet, gymnastics and long distance running [20,21].

A review has found overweight to be inversely related to physical activity among adolescents [22], and psychiatric disorders like mood and anxiety disorders are associated with overweight [23]. Low physical activity is also associated with chronic pain in children [24] and adolescents with psychiatric disorders seem to have a high frequency of chronic pain [25]. Further, lower levels of physical activity are thought to be associated with the use of psychopharmacological treatments [26].

Still, research on the relationship between physical activity and psychiatric symptoms in adolescence is limited, especially in adolescents with psychiatric disorder(s).

The aim of this cross-sectional study was to assess the frequency of physical activity and participation in individual or team sports in adolescence, comparing a psychiatric patient sample with a general population sample. Within the clinical sample we aimed to explore these associations across different psychiatric disorders, and whether physical activity was related to use of psychotropic medication, body mass index (BMI) and chronic pain.

We hypothesized that adolescents with psychiatric disorders would report lower levels of physical activity, yet adolescents with eating or hyperkinetic disorders would report higher levels of physical activity, compared with adolescents from the general population. We also hypothesized that boys would report a higher frequency of physical activity than girls, and that the frequency of physical activity would decrease with age in both sexes. In the clinical sample, we expected to find that low level of physical activity was associated with use of psychotropic medication, as well as high BMI and high level of chronic pain.

## Methods

### Study setting and participants

#### Clinical population sample

The present study was part of the larger Health Survey undertaken at the Department of Child and Adolescent Psychiatry (CAP), St. Olav’s University Hospital, Trondheim, Norway. It was a cross-sectional study of all patients aged 13–18 years who visited the CAP clinic at least once between February 15th, 2009 and February 15th, 2011. Emergency patients were also invited to take part after they were stabilized. Exclusion criteria were: considerable difficulties completing the questionnaire because of inadequate language skills, poor cognitive function or a severe psychiatric state that could not be sufficiently stabilized.

Of 1648 eligible and invited adolescents, 717 (43.5%) participated in the CAP survey. This survey and the representativeness of the sample have been described in detail previously [25].

The present study included 566 adolescents, all of whom met the criteria for at least one psychiatric disorder: 307 girls (54.2%) and 259 boys (45.8%). The age distribution is given in Table 1. A few adolescents (n = 15) were 19–20 years at the time of completing the questionnaire, and in further analysis these are included in the age group 17–18 years.

**Table 1:** The age distribution in the CAP survey and the Young-HUNT 3 survey

	**CAP survey n = 566**		**Young-HUNT 3 survey n = 8173**	
**Age: mean (SD)**	15.68 (1.67)		15.89 (1.74)	
**Age distribution: n (%)**				
**- 13 – 14 years**	227/566 (40.1)		2899/8173 (35.5)	
**- 15 – 16 years**	200/566 (35.3)		2746/8173 (33.6)	
**- 17 – 18 years**	139/566 (24.6)		2528/8173 (30.9)	

#### General population sample

The Nord-Trøndelag Health Study, Young-HUNT 3 (http://www.ntnu.edu/hunt/young-hunt) was carried out from 2006 to 2008. All adolescents aged 13–19 years in the county of Nord-Trøndelag who were at school were invited. Of 10 485 invited, 8200 (78.2%) participated. Some 12-year-old children participated (n = 27), but were excluded due to the low number. A few adolescents (n = 19) were 19–20 years at the time of the study, and in further analysis these are included in the age group 17–18 years. Hence, 8173 were part of this study: 4115 girls (50.3%) and 4058 boys (49.7%). The age distribution is given in Table 1.

### Procedures

Newly referred patients and patients already enrolled at the CAP clinic received oral and written invitations during their first visit after the project started. Parental consent was obtained for participants under 16 years of age while participants aged 16 years and over gave written informed consent to participate. Parents were invited to provide supplementary information and they also gave written informed consent to participate. The participants responded to an electronic questionnaire through a password-protected website. This was done at the clinic, without the presence of their parents. A project coordinator could assist if needed. The parents responded to a shorter questionnaire, either electronically or on paper. Data from the participants were collected from medical records.

In the Young-HUNT 3 survey, a comprehensive questionnaire with a wide range of demographic and health-related items was completed by the students during one school hour. Students who were not present at school on the day of the study could complete the questionnaire at a later clinical examination.

### Measures

#### Medical records

The diagnoses were determined according to the International Statistical Classification of Diseases and Related Health Problems, 10th Revision *(ICD-10)* multiaxial diagnostics (Axes I–IV) [27]. The disorder leading to the present referral, most often the diagnosis requiring the most treatment resources, was set as the main diagnosis on Axis 1. Secondary Axis 1-diagnoses were also registered. Diagnoses were made during ordinary clinical practice by a child psychiatrist or child psychologist after reaching a consensus with other professionals from the multi-disciplinary team. The CAP clinic follows standardized procedures for the assessment and diagnosis of common adolescent psychiatric disorders, including hyperkinetic disorders, autism spectrum disorders (ASD), tic disorders, psychosis, anxiety disorders, depression and eating disorders. The procedures typically require a thorough developmental history, interviews with the adolescents and parents, and the use of rating scales suitable for the presenting problem. The assessment may be supplemented with somatic examination, and possible coexisting disorders are explored.

In this study, we classified the patients according to the main Axis I psychiatric diagnoses (ICD-10 codes are specified in Table 2). These were mood disorders (n = 87, of these 74 had a depressive disorder), anxiety disorders (n = 148), eating disorders (n = 22), ASD (n = 39), hyperkinetic disorders (n = 216) and other disorders (n = 54; a broad spectrum of psychiatric disorders with low frequency).

**Table 2:** Physical activity, sports participation, age, BMI and psychotropic drugs in adolescent psychiatric patients, by psychiatric disorder

	**Total sample n = 566**	**Mood disorders**^**a **^**n = 87**	**Anxiety disorders**^**b **^**n = 148**	**Eating disorders**^**c **^**n = 22**	**ASD**^**d **^**n = 39**	**Hyperkinetic disorders**^**e **^**n = 216**	**Other disorders n = 54**
**Physical activity (n = 561): n (%)**							
**- Low activity**	279/561 (49.7)	53/85 (62.4)	64/147 (43.5)	8/22 (36.4)	22/39 (56.4)	104/214 (48.6)	28/54 (51.9)
**- Moderate activity**	166/561 (29.6)	22/85 (25.9)	49/147 (33.3)	6/22 (27.3)	14/39 (35.9)	64/214 (29.9)	11/54 (20.4)
**- High activity**	116/561 (20.7)	10/85 (11.8)	34/147 (23.1)	8/22 (36.4)	3/39 (7.7)	46/214 (21.5)	15/54 (27.8)
**Individual sports (n = 557): n (%)**	366/557 (65.7)	44/84 (52.4)	105/148 (70.9)	22/22 (100.0)	21/38 (55.3)	142/211 (67.3)	32/54 (59.3)
**Team sports (n = 548): n (%)**	183/548 (32.3)	21/83 (25.3)	48/145 (33.1)	12/22 (54.5)	7/37 (18.9)	78/210 (37.1)	17/51 (33.3)
**Age (n = 566): mean (SD)**	15.7 (1.7)	16.4 (1.6)	15.8 (1.7)	16.3 (1.1)	15.3 (1.5)	15.4 (1.7)	15.3 (1.7)
**BMI (n = 550): mean (SD)**	22.30 (4.49)	23.32 (4.78)	22.57 (4.43)	19.88 (4.03)	21.78 (4.75)	22.09 (4.36)	22.14 (4.44)
**Psychotropic drugs (n = 506): n (%)**	301/506 (59.5)	33/87 (37.9)	40/148 (27.0)	6/22 (27.3)	21/39 (53.8)	175/216 (81.0)	26/54 (48.1)

The numbers in this table, for example n = 561, indicated that 561 of 566 with a psychiatric disorder answered the question about physical activity. 53/85 (62.4), indicated that 53 of 85 with mood (affective) disorders exercised once a week or less, which shows that we had two missing values (n = 87). This applies to the entire table.

^a^ICD-codes F31 – F34, F38 – F39.

^b^ICD-codes F40 – F 44, F48 and F93.

^c^ICD-code F50.

^d^ICD-code F84.

^e^ICD-code F90.

^f^ICD-codes F20 – F21, F28 – F29, F54, F59 – F60, F91 – F92, F94 – F95 and F98.

#### Physical activity

In both the CAP survey and the Young-HUNT 3 survey, self-reported physical activity was assessed by two identical questions from the World Health Organization Health Behaviour in School-Aged Children (HBSC) surveys [28] addressing frequency and amount of time spent on physical activity, outside school. This instrument has previously been validated in the Young-HUNT study cohort [29]. The question regarding frequency was: “Apart from the average school day, how many days a week do you play sports or exercise to the point where you breathe heavily and/or sweat?”. The response options were “Never (1)”, “Less than once a month (2)”, “Not every two weeks, but at least once a month (3)”, “Not every week, but at least once every two weeks (4)”, “One day a week (5)”, “2-3 days a week (6)”, “4-6 days a week (7)” and “Every day (8)”. The question regarding duration was: “Apart from the average school day, how many hours a week do you play sports or exercise to the point where you breathe heavily and/or sweat?”. The response options were: “None (1)”, “About ½ hour (2)”, “About 1–1 ½ hours (3)”, “About 2–3 hours (4)”, “About 4–6 hours (5)” and “7 hours or more (6)”. The frequency question, which inquired about days per week, has been shown to estimate physical activity more precisely than the duration question [29]. For this reason, we chose to use only the frequency question in this study. The answers were recoded into three categories: “low activity” represented “one day a week or less”, “moderate activity” represented “2–3 days a week”, and “high activity” represented “4 days a week or more” [30,31].

Furthermore, participation in sports was assessed with one question: “How often have you done/participated in any of the following activities/sports in the past 12 months?”. The response options were “Never (0)”, “Less than once a week (1)”, “Once a week (2)” and “Several times a week (3)”. Answers from these questions were divided into “No/never (0)”, which entailed response options “0 and 1”, and “Yes (1)”, which entailed response options “2 and 3”. The different sports were divided in two groups: “individual sports“ and “team sports” [31]. “Individual sports” included endurance sports, jogging/race-walking/hiking, strength sports, martial arts, adrenaline sports, esthetics sports and technical sports. Because it was difficult to determine whether the box “other sports” represented “individual sports”, “team sports”, or both, we chose to disregard this group to avoid misclassification.

#### Medication

More than half of the adolescents in the CAP survey used psychotropic drugs (n = 305): anticonvulsants (Anatomical Therapeutic Chemical (ATC) subgroup N03, n = 10), psycholeptics (ATC subgroup N05, n = 42), antidepressants (ATC subgroup N06A, n = 71) and psychostimulants (ATC subgroup N06B, n = 213). In this study we only used two categories for data analyses: “psychotropic medication used” and “no psychotropic medication used”.

#### Chronic pain

Adolescents in the CAP survey were asked to specify if they had experienced headaches or migraines, abdominal pain, or musculoskeletal pain. The frequency of pain in each location was specified as; never/seldom (1), once a month (2), once a week (3), more than once a week (4), or almost every day (5). Chronic pain was defined as pain not related to any known disease or injury, occurring at least once a week in the last 3 months [32]. Prevalence and patterns of chronic pain in the CAP cohort have been reported previously [25].

#### Body mass index

BMI is a proxy for estimating human body fat derived by weight (kg) divided by the square of height (meters) [33].

#### Socioeconomic status

Socioeconomic status was measured using parental level of education; the highest level of education was used to represent the socioeconomic status for the adolescent. In the CAP survey, the parents reported their educational level. In the Young-HUNT 3 survey, Statistics Norway made this information available.

Parental level of education was divided into four categories: 1) less than compulsory school or one to two years in high school (a maximum of 11 years); 2) completed high school and one year education and training after high school (a maximum of 13 years); 3) academy/university for up to and including four years (a maximum of 16 years); 4) academy/university for five years or more, or a PhD (a total of 17 years or more).

### Statistics

Outcome variables were physical activity in three ordered categories (low activity, moderate activity, high activity), individual sports (yes/no) and team sports (yes/no). Differences in proportions were analyzed by Pearson´s chi-squared test, the Wilcoxon-Mann-Whitney test and the Kruskal-Wallis Test. The association between diagnostic groups and each outcome variable was analyzed using ordinal or binary logistic regression. We also carried out analyses adjusting for age and sex as potential confounders, and checked for interactions between sex and diagnostic group. When maximum likelihood estimation (MLE) did not converge, we used Penalized MLE (PMLE, Firth's method) as recommended by Heinze and Schemper [34]. We used ordinal and binary logistic regression to explore possible differences in the risk of low activity between adolescents in the CAP survey and in the Young-HUNT 3 survey. Ninety-five percent confidence intervals (CI) were reported where relevant. Two-sided *P* values of < 0.05 were considered statistically significant. Statistical analyses were done in SPSS 19 (IBM, Chicago, IL, USA), except PMLE, which was done in LogXact10 (Cytel, Cambridge, UK).

### Ethics

In both the CAP survey and the Young-HUNT 3 survey, written informed consent was obtained from adolescents and parents prior to inclusion. Study approval was given by the Regional Committee for Medical and Health Research Ethics (reference number for the CAP survey: 4.2008.1393, for the Young-HUNT 3 survey: 4.2006.250, for the present study: 2011//2061/REK midt).

## Results

Compared to the Young-HUNT sample, a significantly larger proportion of adolescents in the CAP survey reported low levels of physical activity (50% vs. 25%, *P* < 0.001, Table 3). Furthermore, adolescents from the CAP survey participated significantly less in both individual sports (66% vs. 87%, *P* < 0.001) and team sports (32% vs. 61%, *P* < 0.001) than those in the Young-HUNT 3 survey.

**Table 3:** Physical activity, sports participation and BMI in the CAP survey vs. the Young-HUNT 3 survey, by sex

	**CAP survey**				**Young-HUNT study**				
	**Total n = 566**	**Girls n = 307**	**Boys n = 259**	**P Girls vs. boys**	**Total n = 8173**	**Girls n = 4058**	**Boys n = 4115**	**P Girls vs. boys**	**P CAP total vs. Young-HUNT total**
**Physical activity: n (%)**									
**- Low activity**	279/561 (49.7)	152/306 (49.7)	127/255 (49.8)	0.630	1969/8046 (24.5)	1059/4050 (26.1)	910/3996 (22.8)	< 0.001	< 0.001
**- Moderate activity**	166/561 (29.6)	97/306 (31.7)	69/255 (27.1)		2814/8046 (35.0)	1539/4050 (38.0)	1275/3996 (31.9)		
**- High activity**	116/561 (20.7)	57/306 (18.6)	59/255 (23.1)		3263/8046 (40.6)	1452/4050 (35.9)	1811/3996 (45.3)		
**Individual sports: n (%)**	366/557 (65.7)	203/304 (66.8)	163/253 (64.4)	0.561	6749/8026 (84.1)	3535/4055 (87.2)	3214/3971 (80.9)	< 0.001	< 0.001
**Team sports: n (%)**	183/548 (32.3)	91/301 (30.2)	92/247 (37.2)	0.083	4844/7916 (61.2)	2359/4002 (58.9)	2485/3914 (63.5)	< 0.001	< 0.001
**BMI: mean (SD)**	22.30 (4.49)	22.84 (4.76)	21.67 (4.09)	0.001	22.16 (3.83)	22.18 (3.76)	22.13 (3.90)	0.074	0.735

The numbers in this table, for example 279/561 (49.7), indicated that 279 out of 561 adolescents with any psychiatric disorder were physically active one day a week or less, indicating that we had five missing values (n = 566). This applies to the entire table. Also, results from the Mann–Whitney-*U* test apply for the three values of the variable “physical activity”.

In the clinical sample, low levels of physical activity were most frequent among adolescents with mood disorders (62%, Table 2). In contrast, high levels of physical activity were found in 21% of the total sample, with the highest frequency in those with eating disorders (36%). Almost half of the adolescents with hyperkinetic disorders reported low levels of physical activity. Those with mood disorders were less physically active than those with anxiety and eating disorders (*P* < 0.05), and those with eating disorders were also more active than those with ASD (*P* < 0.05). Adjusting for sex, age and SES did not change these associations, and no significant interaction effects of sex with psychiatric disorders were found (data not shown).

Participation in individual sports was reported by 66% of the clinical sample, while 32% participated in team sports. This pattern was generally consistent for all disorders. Adolescents with mood disorders participated less in individual sports than those with anxiety disorders and hyperkinetic disorders, while those with eating disorders participated more than all the other diagnostic groups (*P*-values from 0.0052 to 0.047). Adjustment for sex, age and SES did not change the associations between different psychiatric disorders and participation in individual sports, and no statistical significant interaction effects of sex and psychiatric disorders were found (data not shown). Unadjusted, those with eating disorders participated more in team sports than those with mood disorders, ASD, hyperkinetic and other disorders (*P* < 0.05). When adjusted for sex, age and SES adolescents with eating disorders still participated more in team sports than those with mood disorders, ASD and other disorders (*P* < 0.05), and those with anxiety and hyperkinetic disorders reported higher participation in team sports than those with mood disorders and ASD (*P* < 0.05). We found no interaction effects of sex and psychiatric disorders (data not shown).

BMI was essentially the same (*P* = 0.735) among adolescents in the CAP survey and in Young-HUNT 3 survey (Table 3). However, there were significant differences in BMI between the diagnostic groups (*P* = 0.02) in the CAP survey (Table 2). Adolescents with mood disorders had the highest BMI, followed by adolescents with anxiety disorders. Those with eating disorders had the lowest mean BMI. While we found no evidence of an association between the level of physical activity and BMI in adolescents in the CAP survey (*P* = 0.322), a higher BMI was associated with a lower level of physical activity (*P* < 0.001) in adolescents in the Young-HUNT 3 survey.

Psychotropic drugs were used more frequently by boys (62.9%, n = 165), than by girls (45.0%, n = 138) in the CAP survey (*P* < 0.001), reflecting the higher frequency of hyperkinetic disorders in boys. Overall there was no significant association between use of medication and level of physical activity (*P* = 0.434), and use of stimulants did not differ from the use of other medications, in association with physical activity (*P* = 0.293). Furthermore, use of medication was not associated with BMI in the CAP survey (*P* = 0.295).

Chronic pain was reported by 393 adolescents (70.2%) in the CAP survey [35], but chronic pain was not associated with the level of physical activity (*P* = 0.800).

Girls and boys in the CAP survey did not differ in terms of physical activity levels and participation in individual and team sports (Table 3). Physical activity decreased with age (*P* < 0.001) in the CAP survey, for both individual sports (*P* = 0.061), and team sports (*P* < 0.001). Girls in the Young-HUNT 3 survey reported low levels of physical activity more frequently than boys (26% vs. 23%, respectively, *P* < 0.001, Table 3). Additionally, girls in the Young-HUNT 3 survey participated more in individual sports than boys (87% vs. 81%, *P* < 0.001). Also, in the Young-HUNT 3 survey physical activity decreased with age (*P* < 0.001), for both individual sports (*P* < 0.001) and team sports (*P* = 0.006).

Girls and boys in the CAP survey reported lower levels of physical activity than adolescents in the Young-HUNT 3 survey (*P* < 0.001, Table 3). Girls in the CAP survey also participated significantly less than girls in the Young-HUNT 3 survey in both individual sports (67% vs. 87%, *P* < 0.001) and team sports (30% vs. 60%, *P* < 0.001). Similarly, boys in the CAP survey participated less in individual sports (65% vs. 81%, *P* < 0.001) and team sports (37% vs. 64%, *P* < 0.001) than boys in the Young-HUNT 3 survey.

Adolescents in the CAP survey had a three-fold increased crude ratio for reporting low levels of physical activity compared to adolescents in the Young-HUNT 3 survey (Table 4). The odds ratio (OR) remained virtually unchained after adjustment for sex, age and SES (OR = 3.00, 95% CI 2.48–3.62). Adolescents in the CAP survey also participated less in individual sports (OR = 2.76, 95% CI 2.30–3.32) and team sports (OR = 3.15, 95% CI 2.62–3.78). When adjusted for sex, age and SES, the estimates remained approximately the same for both individual sports (OR = 2.89, 95% CI 2.33–3.60) and team sports (OR = 3.36, 95% CI 2.71–4.17).

**Table 4:** Physical activity and sports participation in the CAP survey vs. the Young-HUNT 3 survey

	**Low activity**			**Not participating in individual sports**			**Not participating in team sports**		
**Overall**	**n**	**OR (95% CI)**	***P***	**n**	**OR (95% CI)**	***P***	**n**	**OR (95% CI)**	***P***
**Unadjusted**									
*CAP survey*	8607	2.91 (2.48 to 3.42)	< 0.001	8583	2.76 (2.30 to 3.32)	<0.001	8464	3.15 (2.62 to 3.78)	<0.001
**Adjusted separately for**									
*Sex*	8607	2.89 (2.46 to 3.40)	< 0.001	8583	2.83 (2.35 to 3.41)	<0.001	8464	3.13 (2.60 to 3,76)	<0.001
*Age*	8607	2.99 (2.54 to 3.52)	< 0.001	8583	2.79 (2.32 to 3.36)	<0.001	8464	3.35 (2.79 to 4.04)	<0.001
*Socioeconomic status*	8370	2.83 (2.34 to 3.41)	< 0.001	8345	2.78 (2.24 to 3.45)	<0.001	8230	3.35 (2.79 to 4.04)	<0.001
**Adjusted for all**	8370	3.00 (2.48 to 3.62)	< 0.001	8345	2.89 (2.33 to 3.60)	<0.001	8230	3.36 (2.71 to 4.17)	<0.001

Ordinal regression was used in “low activity”, while binomial logistic regression was used in “not participating in individual sports” and “not participating in team sports”.

## Discussion

Adolescents with a psychiatric disorder had a three-fold increased risk of lower levels of physical activity, and also approximately a three-fold increased risk of not participating in team and individual sports, compared with adolescents in the general population. Those with mood disorders and ASD were the most inactive, and those with eating disorders the most active, with the same pattern in individual and team sports. Level of physical activity was not related to use of psychotropic medication, BMI or level of chronic pain. Two other studies have found a similar result in adults with severe psychiatric disorders (schizophrenia, schizoaffective disorder, bipolar disorder or major depression) compared with healthy controls [2,36]. This is the first study to replicate these findings in a clinical adolescent sample with less severe psychiatric conditions.

In our study, more than 60% of adolescents with mood disorders and 40% of those with anxiety disorders reported low levels of physical activity. These numbers correspond with other findings of low levels of physical activity in adults and adolescents with depression and anxiety [37-40]. Although little is known about the level of physical activity across psychiatric disorders in adolescents, previous findings have shown an association between low levels of physical activity and symptoms of depression in adolescents [41]. According to a review, there is an inverse relationship between physical activity, particularly sports participation, and level of depressive symptoms [42]. The psychopathology of some psychiatric disorders, such as depression and anxiety, are associated with a sedentary lifestyle in psychiatric patients [43]. Adolescents with mood or anxiety disorders might participate less in physical activity because of a lack of interest, feeling tired or avoiding the social part of physical activity and sports participation [43]. Recent findings also indicate that untreated depression hinders the positive effects of physical activity in adults [44]. Some adolescents in our study may have had untreated depression, which may have contributed to low levels of physical activity. A low level of physical activity and social isolation can in turn increase depressive and anxious symptoms, creating a vicious circle. Previous reports suggest an association between early stress and hyperactivity of the hypothalamic pituitary adrenal (HPA) axis in mood and anxiety disorders, resulting in a permanently unstable and dysfunctional HPA axis [45]. In general, hyperactivity of the HPA axis is also associated with sedentary behavior [46].

As expected, in the clinical sample adolescents with eating disorders had the highest frequency of physical activity and participation in sports. Most of these adolescents were girls. They may experience the “female athlete triad syndrome”: disordered eating, cessation of the menstrual cycle and osteoporosis [47]. If an athlete is suffering from one element in the triad, it is likely that she is suffering from the two other components. The disordered eating involves leptin dysregulation: upon severe food restriction, a low level of leptin stimulates physical activity (seeking food) [48], and with weight gain leptin levels increase, and the need for physical activity declines [49].

In keeping with our hypotheses, adolescents with ASD had low levels of physical activity, and of all the diagnostic groups, the lowest participation in team sports. ASD are characterized by difficulties in social interaction and in verbal and non-verbal reciprocal communication [50], skills that are especially important in team sports. Furthermore, 82% of the adolescents in the group with ASD had Asperger’s syndrome. This disorder is often associated with marked clumsiness, which entails difficulties walking, crawling and running [51]. This further supports our findings. Not being able to participate in physical activity, team sports in particular, might be particularly worrying for these adolescents, as they miss out on the social aspects of physical activity that contribute to the positive effects on mental health [52].

We hypothesized that adolescents with hyperkinetic disorders would report high frequencies of physical activity and sports participation, given hyperactivity is a core symptom of their disorders. However, approximately half of them reported low levels of physical activity, and more than half of them reported non-participation in team sports. One explanation might be that the activity drive is expressed in ways other than purposeful physical activity and participation in sports, that is, as more inappropriate hyperactivity. Researchers have found that boys with ADHD exhibit higher levels of aggression and emotional reactivity than boys without ADHD, in sport settings [53]. Furthermore, their inattention and impulsivity can also be a challenge in sports where prolonged attention and cautious behavior are needed. Some findings indicate that people with hyperkinetic disorders often have trouble with their working memory, especially visuospatial working memory [54], which may be a contributing factor for non-participation in team sports. Taking medication for hyperkinetic disorders might affect performance in a positive way, helping the adolescents stay more focused [55]. In our study, we found no differences in the levels of physical activity, as measured in this study, between those with hyperkinetic disorders who took medication, and those who did not.

Some of the adolescents with a psychiatric disorder used other prescribed medications for treatment of that disorder. The sedative effects of psychopharmacological treatments are associated with a sedentary lifestyle in psychiatric patients [26], which may result in lower levels of physical activity. However, we found no statistical differences in physical activity between adolescents who took medication and those who did not. Furthermore, some medications can cause weight gain through disturbed appetite, slow metabolism or bloating, which in turn can affect the level of physical activity [22]. However, adolescents in our clinical sample had only slightly higher BMIs than adolescents in the normal population sample, and there was no association between BMI and the level of physical activity in adolescents with a psychiatric disorder. Hence, in our sample, weight did not seem to affect the level of activity in those with a psychiatric disorder. Furthermore, chronic pain is known to negatively affect levels of physical activity [24], and adolescents with a psychiatric disorder usually have more chronic pain than those in the general population [56,57]. Although the frequency of chronic pain was high in our study, we found no association between the level of physical activity and chronic pain.

Overall, girls in the Young-HUNT 3 survey were less physically active than boys, more specifically they participated more in individual sports but less in team sports, which confirms previous studies [7,58]. In contrast, in the CAP survey, interaction tests indicated that there were no sex differences in physical activity or participation in sports.

In summary, besides the diagnostic categories and their symptoms discussed above, we did not identify any single factor associated with psychiatric disorder that could explain the low physical activity in the clinical sample. We cannot rule out residual confounding: the possibility that other factors that were not accessible in this study had an effect on physical activity levels.

Although the evidence is limited in adolescents, positive effects of physical activity and involvement in sports on symptoms of depression and anxiety have been reported [43,59]. Possible mechanisms might be an increase in serotonin and endorphin levels, producing analgesia and a sense of well being, thereby providing an effect similar to that of antidepressants [60,61]. It has been shown in adults that physical activity can increase the synthesis of new hippocampal neurons, which induces a mood-elevating effect [62]. Physical activity also increases levels of dopamine, increasing the feeling of motivation, and acting as a positive reinforcement to continue with the physical activity [63]. The psychological feeling of competence and increased self-esteem [43], may reduce the level of depression and anxiety [64]. Being physically active might also enhance peer relationships, which in turn may contribute to positive health-related outcomes [65].

Given today’s knowledge about the positive effect of physical activity on both physical and mental health, it is imperative to identify adolescents at risk, or who already have a psychiatric disorder, and initiate interventions to increase physical activity as part of their treatment. In particular, adolescents with hyperkinetic disorders need to take part in appropriate activity settings where their level of activity can be seen as a strength. Helping adolescents with ASD to participate in a sport or activity they master, despite the clumsiness, is critical in preventing loneliness and other core problems they encounter. This is essential, not just to prevent the negative consequences, but also to promote the positive effects of physical activity as an additional treatment for psychiatric disorders. Breaking the association between psychiatric disorders and low physical activity is essential, and these findings indicate the period of adolescence as a crucial time window within which to do that.

Our study had several strengths: it included a relatively large clinical sample, and employed both self-report measures and psychiatric diagnoses validated by a child psychologist or psychiatrist. The participants were representative with regard to reasons for referral, coded according to a national classification system of suspected disorders. Unlike previous research, we investigated physical activity across diagnoses to discover associations between psychiatric diagnostic groups and activity levels. We also compared the results with a general population sample derived from the Young-HUNT 3 survey, which increased the relevance and generalizability of the results.

Some limitations of this study need to be taken into account. This is a cross-sectional study, and the temporal nature of the association cannot be elucidated. Only 43% of the eligible and invited patients participated in the CAP survey, which means that this study should be replicated. Even though the reason for referral highlights the main problem area to be examined, it may not coincide completely with the final diagnosis. Hence, our results may not be applicable to other populations and study settings. Inter-rater reliability for diagnostic assessment was not assessed. However, the diagnoses were made by an experienced child psychiatrist or psychologist after consensus discussion with professional co-workers in the multi-disciplinary team, according to national guidelines and procedures. We measured physical activity in adolescents by self-reported questionnaires, and this retrospective report may have been influenced by recall bias, which in turn could have led to an under- or overestimation of physical activity.

In conclusion, adolescents with a psychiatric disorder reported low levels of physical activity. Compared with adolescents in the general population, they had a three-fold increased risk of lower physical activity, similar for not participating in individual and team sports. There were no sex differences in the level of physical activity in the CAP survey. The findings underscore the importance of assessing physical activity in adolescents with psychiatric disorders, and of providing early intervention to promote both mental and physical health in this early stage of life.

## Competing interests

The authors declare that they have no competing interests.

## Authors’ contribution

WLM designed and drafted the manuscript with guidance from MSI and OB. WLM conducted the analysis and interpreted the data with guidance from SL. MSI, OB, and SL revised the manuscript for important intellectual content. All authors gave final approval on the version to be published.
